# Medieval Horse Stable; The Results of Multi Proxy Interdisciplinary Research

**DOI:** 10.1371/journal.pone.0089273

**Published:** 2014-03-26

**Authors:** Miroslav Dejmal, Lenka Lisá, Miriam Fišáková Nývltová, Aleš Bajer, Libor Petr, Petr Kočár, Romana Kočárová, Ladislav Nejman, Michal Rybníček, Zdenka Sůvová, Randy Culp, Hanuš Vavrčík

**Affiliations:** 1 Archaia Brno, o. p. s., Brno, Czech Republic; 2 Institute of Geology AS CR, v. v. i., Prague, Czech Republic; 3 Institute of Archaeology of AS CR in Brno, v. v. i., Brno, Czech Republic; 4 Faculty of Forestry and Wood Technology, Mendel University in Brno, Brno, Czech Republic; 5 Department of Archaeology, University of Western Bohemia, Pilsen, Czech Republic; 6 Centre for Applied Isotope Studies, University of Georgia, Athens, Georgia, United States of America; 7 School of Social Science, University of Queensland, St Lucia, Australia; Manchester Institute of Biotechnology, United Kingdom

## Abstract

A multi proxy approach was applied in the reconstruction of the architecture of Medieval horse stable architecture, the maintenance practices associated with that structure as well as horse alimentation at the beginning of 13^th^ century in Central Europe. Finally, an interpretation of the local vegetation structure along Morava River, Czech Republic is presented. The investigated stable experienced two construction phases. The infill was well preserved and its composition reflects maintenance practices. The uppermost part of the infill was composed of fresh stabling, which accumulated within a few months at the end of summer. Horses from different backgrounds were kept in the stable and this is reflected in the results of isotope analyses. Horses were fed meadow grasses as well as woody vegetation, millet, oat, and less commonly hemp, wheat and rye. Three possible explanations of stable usage are suggested. The stable was probably used on a temporary basis for horses of workers employed at the castle, courier horses and horses used in battle.

## Introduction

Despite the fact that horses were a common part of the Medieval culture, there is a dearth of information about horse husbandry in the Central European Middle Ages. Occasional references can be found in written documents [1]–[3] as well as in archaeological research [4]–[5], but the identification of architectonical structures is usually very difficult and stabling deposits are not commonly preserved.

Important information about stable deposits can be inferred from microstratigraphic studies [6]–[9] Floor deposits in particular can be a source of high-value information. Variations in floor residues are being profitably examined in order to understand uses of space and the nature of activities in a settlement [7] [10]–[17], but archaeological stratigraphies that may contain floors are usually difficult to identify when not composed of lithologically varied materials. One of the well recognizable types of floor deposits are those composed of stabling deposits. Micromorphological research of stable floor deposits has been reported from different environments but such studies are rare. An experimental stable was studied in Butser ancient farm [18].

A unique opportunity to perform interdisciplinary research including a classic archaeological approach, combined with geology and micromorphology, bioarchaeology and isotope studies of a well preserved Medieval stable presented itself in 2009, when during rescue excavations in Veselí nad Moravou, occupation deposits were found within a bailey dated to an early phase of castle construction. This paper discusses what Medieval stable architecture looked like and how the stable was maintained. Both, the specific diet of horses which occupied the stable during the last phase of its use, and the vegetation growing in the castle surroundings are discussed in this paper.

## The study site: Medieval bailey in Veselí nad Moravou, Czech Republic

**Figure 1:**
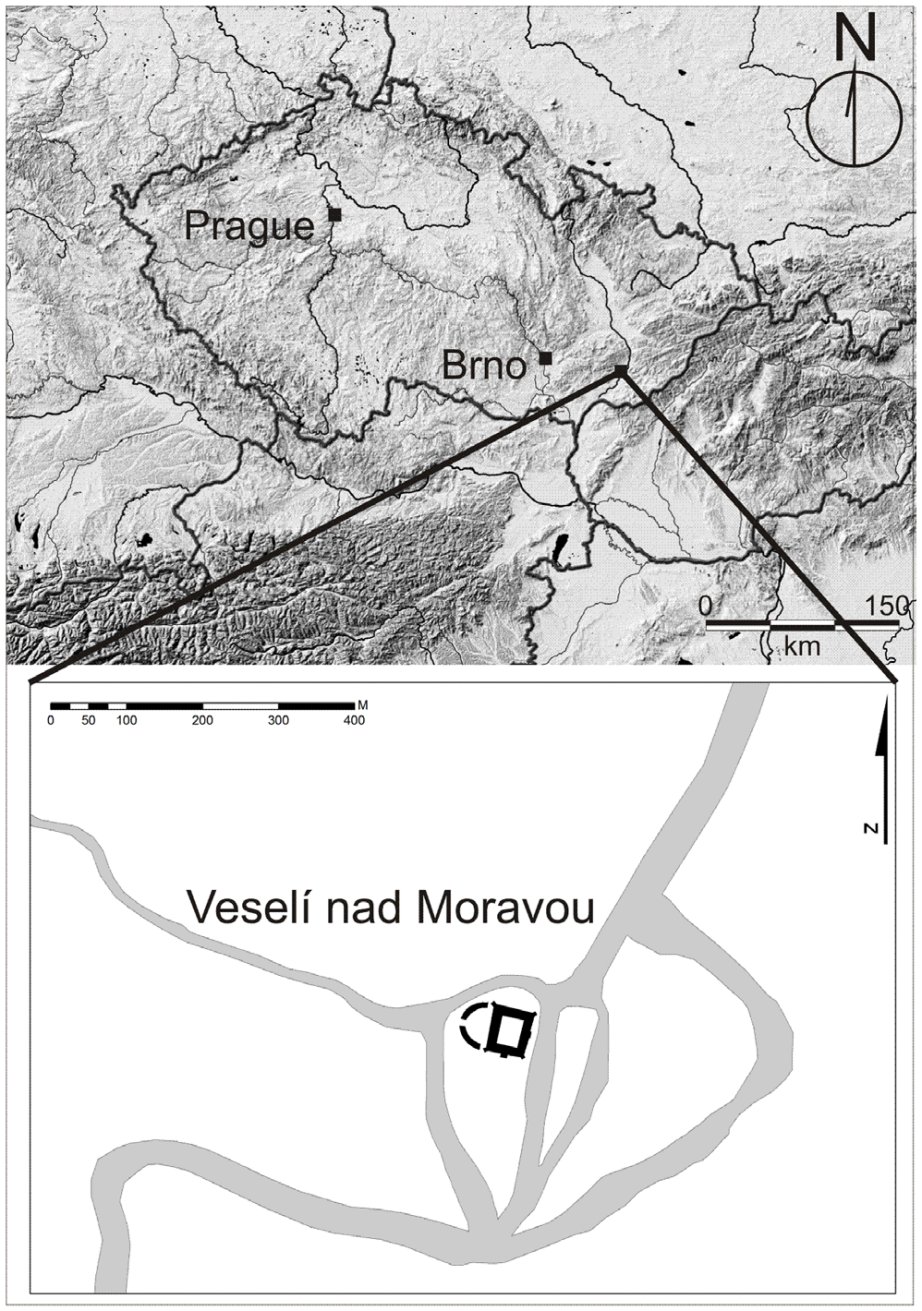
The location of Veselí nad Moravou within the context of Central Europe and within the local geomorphology.

**Figure 2:**
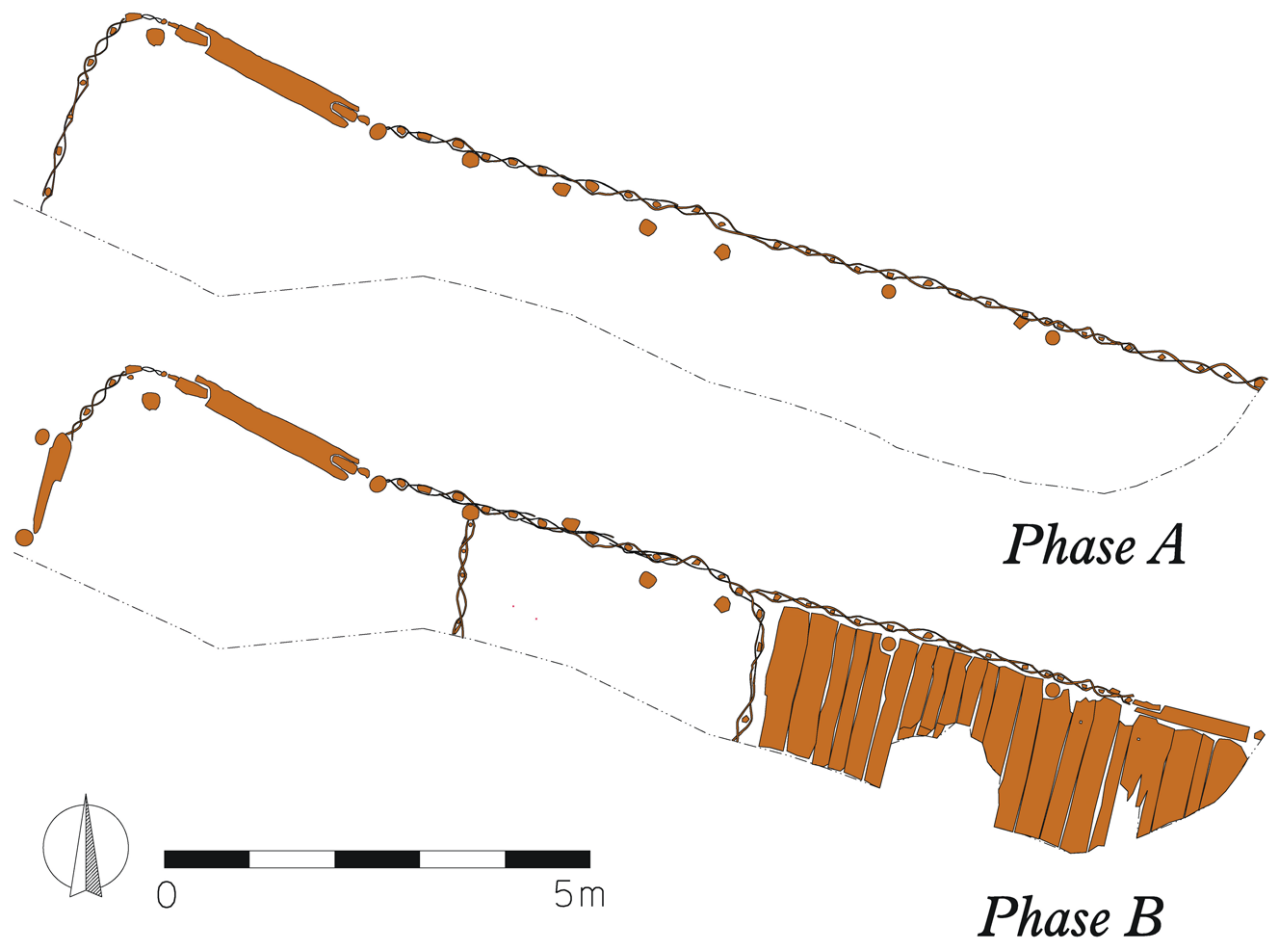
Archaeological evidence of horse stable.

Veselí nad Moravou is situated in the southeastern part of the Czech Republic in South Moravia. The study site is located on the right bank of the Morava River (Fig. 1). The archaeological rescue research was performed during renovation of the castle in 2008–2010 [19]. Within the castle grounds, located features include a nobleman residence and a wooden bailey which was separated from the castle itself by a moat. Also, well preserved occupational deposits covering an area of 500 m^2^ were identified. These deposits were 1.6 meters thick and consisted mainly of wooden chips and organo-mineral material. Only the uppermost part of the occupational deposit was destroyed by decomposition typical for dry open air environments. In total, 16 wooden objects were detected, but the function of these structures was proposed in only three cases. One of them was interpreted as a bakery, the second one as a hayloft and the third one, discussed in this paper, as a horse stable (Fig. 2) [19]. The reason why the structure was interpreted as a horse stable is mainly due to the presence of well preserved stabling material containing horse hairs as well as artefacts which are typically used in the context of horse husbandry.

## Research methods

### 1. Ethics statement

We declare that no living animals were used in this research. The samples used for zooarchaeological research and isotope studies were collected from an archaeological context dated to the 13^th^ century. The archaeological company conducted the excavations under a government permit and following the heritage law of Czech Republic (No. 20/1987), all excavated material including the osteological material is interpreted as archaeological material so no further permits are required for the presented study.

### 2. Archaeology

The excavation area was divided into 5×5 squares. Those squares were then excavated in 2×2 subsquares and a control section 0.5 m wide was left unexcavated along each square. In some cases, sectors were selected irregularly, based on specific contexts. This method of excavation was selected for better orientation in a stratigraphically difficult situation. The excavation was systematically documented with drawings and photographs. Coordinates of archeological features and artefacts were recorded. The Harris matrix showing the connections between different contexts was applied. Dominant structures were differentiated from other contexts (layers, constructions and ditches). Finally, recovered artefacts and their spatial connections were analysed.

### 3. Sedimentological description, micromorphology

**Figure 3:**
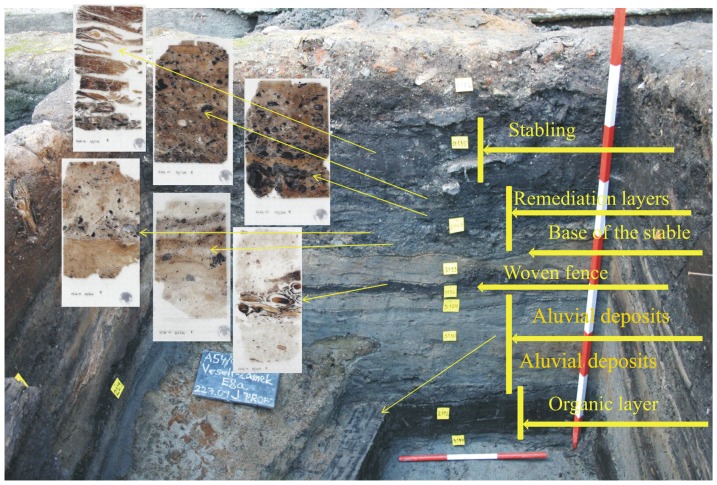
Micromorphological sampling position with the sedimentary facies interpretation.

The stratigraphic section which included the stable infill and underlying sediment (Fig. 3) was approximately 80 cm thick. It was sedimentologically logged and lithological changes were described. Seven micromorphological samples were then collected from the studied section based on visible lithological changes, placed into plastic Kubiena boxes (5×9 cm) and prepared according to standard procedure by Julie Boreham in Reach (www.earthslides.com). Thin sections were studied under a binocular and polarising microscope at different magnifications and described according to Bullock et al. [20] [21] and Stoops [22] [23].

### 4. Pollen and phytolith analysis

Four samples for pollen and phytolith analyses were collected from the top 20 cm of the stable infill, i.e. from the horse stabling material. Preparation followed the standard acetolysis method [24]. For phytolith samples, the wet oxidation method was used [25]. Pollen grains and phytoliths were distinguished using published guides [26]–[30]. Each sample contained a minimum of 500 grains or fragments. The pollen diagram includes phytoliths and was plotted in POLPAL program for Windows [31] including numerical analyses (Rarefraction, Conslink, PCA) for the visualization and the interpretation of pollen data.

### 5. Macroremains and anthracology

Macroremains and anthracological analyses were performed on samples from different parts of the stable infill. In total, 7 samples were processed, three from the horse stabling and four from the waste material underlying the horse stabling. Extraction of macrofossils from the soil material followed the standard flotation and wet sieving procedure [32], using staggered sieves with 0.25 mm mesh. The dried samples were then sorted under light microscope at ×l0 to ×15 magnifications. Seed atlases by Cappers et al. [33] [34] were used for plant macro-remains determination. Fresh refractive surfaces of wooden fragments and charcoal were analysed under a light microscope at 50×, 100× and 200× magnifications. The number of fragments was counted. Identification followed mainly Schweingruber [35] and a web key [36].

### 6. Isotopes

Six samples of horsehair were divided into three centimeter (cm) sections (Tab. 3), and analyzed for their respective δ13C and δ15N values. The analyses were performed at the University of Georgia's Center for Applied Isotope Studies, Athens, Georgia, USA, using standard procedure. Approximately 200–600 micrograms of horsehair from each sample was analysed. Two interpretation diagrams based on [37]–[40] were constructed (Fig. 8 and 9) and used in the general interpretations.

**Table 3:** δ^13^C and δ^15^N values obtained from horse hair samples.

Sample	Lot	δ^13^C	±1 σ	δ^15^N	SD
**Horse hair**	K228/1	−21.79	0.21	6.46	0.20
**Horse hair**	K228/2	−23.57	-	4.48	**-**
**Horse hair**	1268/1	−23.40	0.35	7.84	1.21
**Horse hair**	1268/2	−22.39	0.18	7.25	0.34
**Horse hair**	1268/3	−22.54	0.20	6.62	0.16
**Horse hair**	1268/4	−22.97	0.16	7.30	0.30
**Horse hair**	3318/1	−23.18	0.21	6.05	0.74
**Horse hair**	3318/2	−24.09	0.17	5.07	0.29
**Horse hair**	3318/3	−23.23	0.78	5.15	0.64
**Horse hair**	3318/1B	−23.66	0.48	6.71	0.46
**Horse hair**	3318/2B	−22.69	0.11	6.67	0.17
**Horse hair**	3318/3B	−22.60	0.04	6.95	0.11
**Horse hair**	3318/4B	−22.38	0.08	6.90	0.01
**Horse hair**	3318/5B	−22.42	0.09	6.88	0.11
**Horse hair**	3318/6B	−22.32	0.03	6.39	0.36
**Horse hair**	3318/7B	−22.37	0.19	4.16	0.43
**Horse hair**	K1268/1B	−22.51	0.44	5.94	0.21
**Horse hair**	K1268/2B	−22.77	0.06	5.66	0.01
**Horse hair**	K1268/3B	−23.60	0.17	9.17	0.79
**Horse hair**	K228/1B	−22.93	0.90	5.54	1.27
**Horse hair**	K228/2B	−22.48	0.14	4.37	0.10

### 7. Dendrochronology and dendrology

Samples for dendrochronological analysis from separate structures identified within the bailey were collected using a drill. The analysis was performed in correspondence with standard dendrochronological methodology [41]. The average age was counted from 6 samples taken within bailey structures. Samples labelled as 110 and 111 come from the stable material. Dendrological analyses of the stable structure were performed using standard identification keys.

### 8. Zooarchaeology

Bone fragments were collected and bagged during the archaeological excavation. The identification was performed using standard zooarchaeological keys. The osteological material is stored in the depository of Archaia o.p.s. company, Bezručova 15, 602 00, Brno.

## Results

### 1. Archaeological evidence of horse stable

The horse stable was 14×2.5 meters in size, but may have originally been larger (its original size has not been determined). It was excavated in the southeastern part of what is currently the castle courtyard. Parts of the building were obliterated by later Medieval activities and construction works in the 1990s. Two construction phases designated as A and B were identified on the basis of different construction features such as walls, pillars and floors (Fig. 2).

Rough-hewn girders 0.06×0.04 m in size were used during construction phase A. The space between these girders was filled with a hurdle fence and covered by a thick layer of daub from both sides. The roof of the building was supported by 0.16 m thick pillars located regularly every two meters along the walls. The 1.6 m wide relict of an entrance was located in the northern part of the building. The entrance structure consisted of a pair of vertical window frames with an added doorstep. Divisions inside the stable were not identified for this construction phase.

During construction phase B the building was completely rebuilt. The former building was destroyed and only the foundations, which were re-used, remained in their original position. The construction technique was very similar to the previous one. New walls mirrored the shape of the former walls and the entrance located in the northwestern part of the building was replaced by a new entrance located in the same position and within the same construction proportions. In contrast to the original building, the interior of the new building was divided by two partition walls. The first wall divided the eastern room (approx. 6 m^2^ in size). The partition wall was constructed in the same way as the outside wall and a hurdle fence was also present. The eastern room had a wooden floor consisting of rough-hewn, criss-crossing planks up to 0.4 meters wide. The 1 meter wide entrance was located in the northern section and consisted of double window frames and a doorstep. The second partition wall, 3.5 metres long, divided the central room. The partition wall was constructed as a hurdle fence and was not joined to the outside wall. It appears that a 1 meter wide entrance facing west was also added. All the doorsteps were raised 0.25 m above the floor. The organic layers deposited on the floor of the stable originating in the second construction phase of contain evidence of horse presence with horseman equipment such as horse shoes, bridle-bits, currycomb, buckles and spurs. Two complete sickles were also recovered.

### 2. Sedimentology and micromorphology of the deposits

#### 2.1. Fluvial deposits and trampling periods

**Figure 4:**
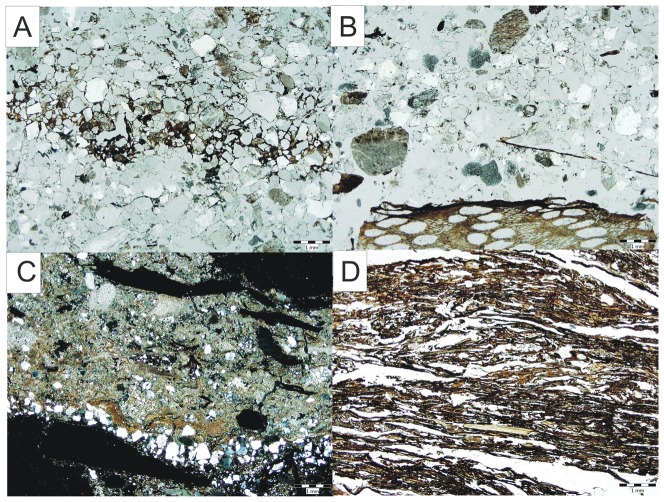
Characteristic micromorphological features in studied section. A - Fluvial deposits with trampling periods and thin layers of decomposed organic matter; B - Fence relict composed of undecomposed organic matter followed by the last phase of inundation; C - Infilling of the stable composed of domestic waste; D - Horse stabling composed of partly decomposed organic matter and mineral components.

The underlying material of the horse stable consists of 10 cm thick, partly decomposed organic material (wood) followed by a 30 cm thick fluvial deposit of grey colour consisting of sands and silts (Tab. 1). The fluvial deposition of sands ends with a layer of organic material, macroscopically identified as part of a collapsed hurdle fence followed by a thin layer of fluvial sands. The organic matter of this structure is not decomposed as seen in Fig. 4B. The sandy material above the fence differs somewhat from the sandy material below. Repeatedly appearing thin, dark brown layers composed of decomposed organic material were identified (Fig. 4A). Those organic intercalations document the phases when the area was not inundated by water and was probably being trampled by humans. The thick fluvial section ends with a partly decomposed organic material macroscopically identified as a relict of a hurdle fence, followed by yellowish sandy material 5 cm thick (Fig. 3).

**Table 1:** Micromorhological description of different lithological contexts.

Context	Description
4.2.1. Fluvial deposits with trampling periods	Moderately sorted subangular to subrounded clasts mainly composed of quartz between 0.2 to 1 mm in size. The prevailing types of pores are simple packing voids and the microstructure of these deposits is porphyric with monic related distribution. Chitonic distribution is associated with decomposed organic matter (Fig. 4 A). Bioturbation was not observed. Charcoal or partly decomposed organic matter occasionally occurs. The main postdepositional process is leaching.
4.2.2. Infill of the horse stabling structure composed of domestic waste	Poorly sorted material composed of charcoal, quartz grains, burned bones, ceramic fragments, partly decomposed organic matter and matrix rich in micritic calcium phosphate compose the infilling of horse stable structure and was interpreted as domestic waste coming mainly from ovens (Fig. 4 C). Its microstructure is porphyric, with porphyric related distribution of angular to subrounded clasts of varied origin and with rare occurrence of compound packing voids. Leaching and accumulations of phosphate rich solutions are visible within the matrix. No bioturbation was observed.
4.2.3. Horse stabling	The horse stabling is composed mainly of partly decomposed organic material, and a large number of horse hairs. The organic material is visibly degraded in the uppermost part of the section. Stable crust is microscopically composed of laminated plant fragments embedded in a calcium phosphate-rich, autofluorescent (blue light) cement of hydroxyapatite (Fig. 4D).

#### 2.2. Infill of the horse stabling structure composed of domestic waste

The basal layer consists of 15 cm thick occupation deposits composed of construction material, charcoal, decomposed organic matter and ashy material (Tab. 1, Fig. 4C). Layering is macroscopically visible within this layer (Fig. 2). The material in each layer differs somewhat in grain size, the amount of daub and ceramic sherds. Within this stratum, visible minute layering corresponds to phases of accumulation. These individual sublayers are composed of different types of material and the grain size varies considerably, but differences in lithological composition are small.

#### 2.3. Horse stabling

The section and the infilling of the horse stable is capped by well preserved horse stabling, i.e. organic material deposited during the last usage of this structure (Tab. 1, Fig. 4D). Horsehair is common in this layer but it is difficult to identify and is observed mainly as round-shaped cross sections (Fig. 10).

### 3. Pollen and phytolith analyses

**Figure 5:**
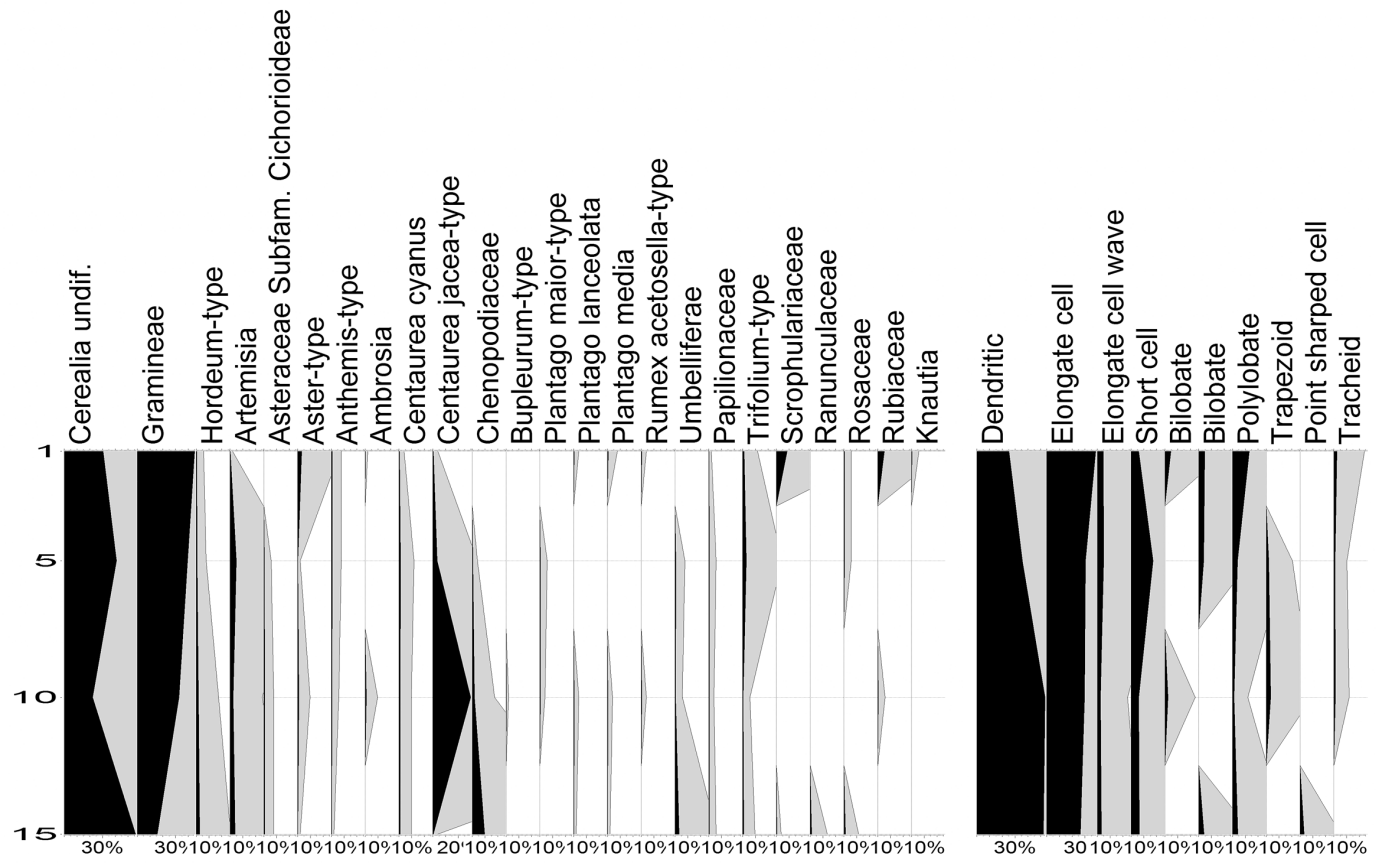
Pollen and phytolith diagram from Veselí nad Moravou Medieval stabling.

Pollen spectra found in the 20 cm thick layer of horse stabling include mainly herbs. Tree palynomorphs are very rare. Most of the herb palynomorphs are wild grasses. Intestinal parasites were not recorded, except for an Ascaris spore in a sample located 10–15 cm below the surface (Fig. 5).

Phytolith spectra are relatively uniform with low type diversity. Dendritic-type phytoliths occur most frequently and elongate-type phytoliths have the second highest frequency. Based on the abundance of Dendritic-type and Polylobate-type types, most of the phytoliths come from wild grasses of subfamilie Poideae. Panicoideae grasses (Bilobate-type) are rare; this type is likewise rare in present day vegetation of Southern Moravia. Phytoliths from trees and cereals were not recorded. The phytolith spectra reflect meadow vegetation where wild grasses and sedges dominate.

### 4. Macroremains and anthracological analyses

**Figure 6:**
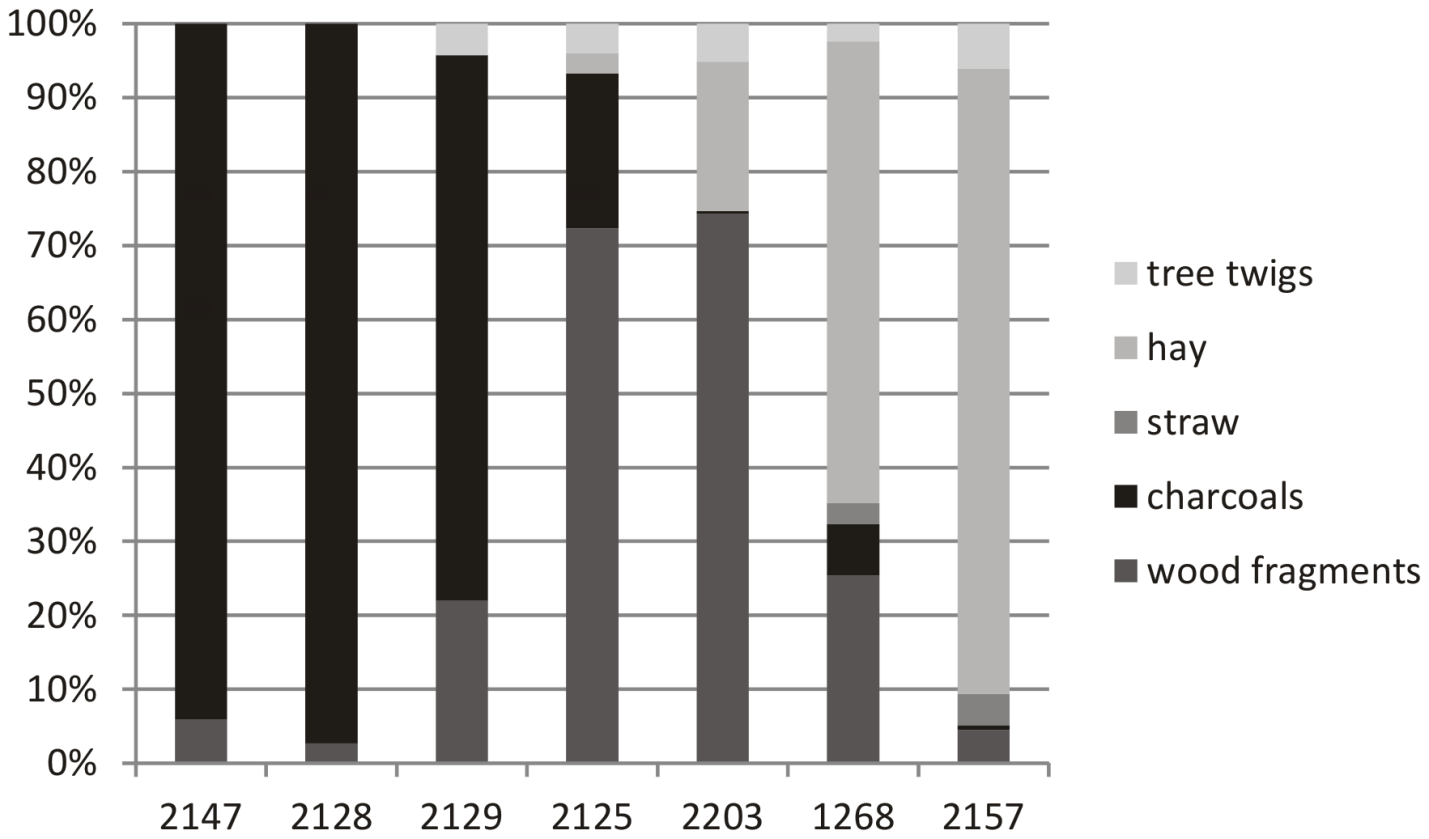
The proportion of macroscopically identifiable components (hay, straw, wood annual shoots). Samples of archaeological contexts 2147, 2129 and 2128 are from the waste aggradation while the samples of the contexts 2157, 2203, 2125 and 1268 are from the stabling.

**Figure 7:**
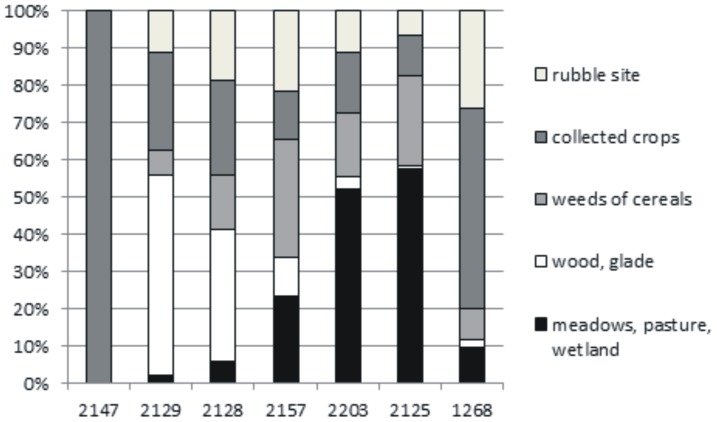
Ecological groups identified within the studied samples (samples of archaeological contexts 2147, 2129 and 2128 are from waste aggradation while samples 2157, 2203, 2125 and 1268 are from stabling.

Macroremains and anthracological spectra identified within the Medieval stable infill in Veselí nad Moravou are quite heterogeneous (Fig. 6 and Fig. 7, tab. 2). Samples from the stable have the highest concentration of macroremains (compared to other units) and lower concentrations of wood fragments and charcoal (Fig. 6). The proportion of individual ecological groups which could be significant for stabling (the concentration of wetland species) or feeding (the concentration of crop plants) varies greatly (Tab. 2). Also, the proportion of macroscopically identifiable components (hay, straw, wood annual shoots) varies significantly in the samples (Fig. 6). After examining 2150 pieces of plant macroremains (mainly seeds and fruits), 104 taxons of higher plants were identified and divided into 5 ecological groups according to their ecological requirements known from recent times (Tab. 2). Those groups are: 1) meadows, pasture, wetland; 2) cereal weeds; 3) economic crops; 4) ruderal plants; 5) wood, glade.

**Table 2:** The types and numbers of macroremains of different contexts (samples of archaeological contexts 2147, 2129 and 2128 come from the remediation layer while samples 2157, 2203, 2125 and 1268 come from stabling).

	context	waste aggradation					horse stabling		
	context number	2147	2129	2128	2203	2157	1268	2125	total
Type of plants	meadows, pasture, wetland		6	4	80	125	274	65	554
	wood, glade		158	25	35	8	4	13	243
	weeds of cereals		19	10	107	41	116	56	349
	collected crops	2	77	18	44	39	51	358	589
	rubble site		33	13	73	27	31	176	353
	total amount	2	293	70	339	240	476	668	2088

The remediation layers in the stable commonly contained charcoal fragments providing clues to wood vegetation in the surrounding area. The most common taxa are typical for oak forests and oak-hornbeam forests including hardwood forests of lowland rivers (*Quercus robur*, *Fraxinus angustifolia subsp. danubialis*, *Fraxinus excelsior*, *Acer campestre*, *Ulmus laevis*). The species typical for willow-poplar forests (*Populus nigra*, *Populus alba*, *Salix alba*, *Salix* fragilis) and alder forests (*Alnus glutinosa*) were present in lower quantities. Charcoal fragments of beech were very rare.

### 5. Isotope analysis of horse hair

**Figure 8:**
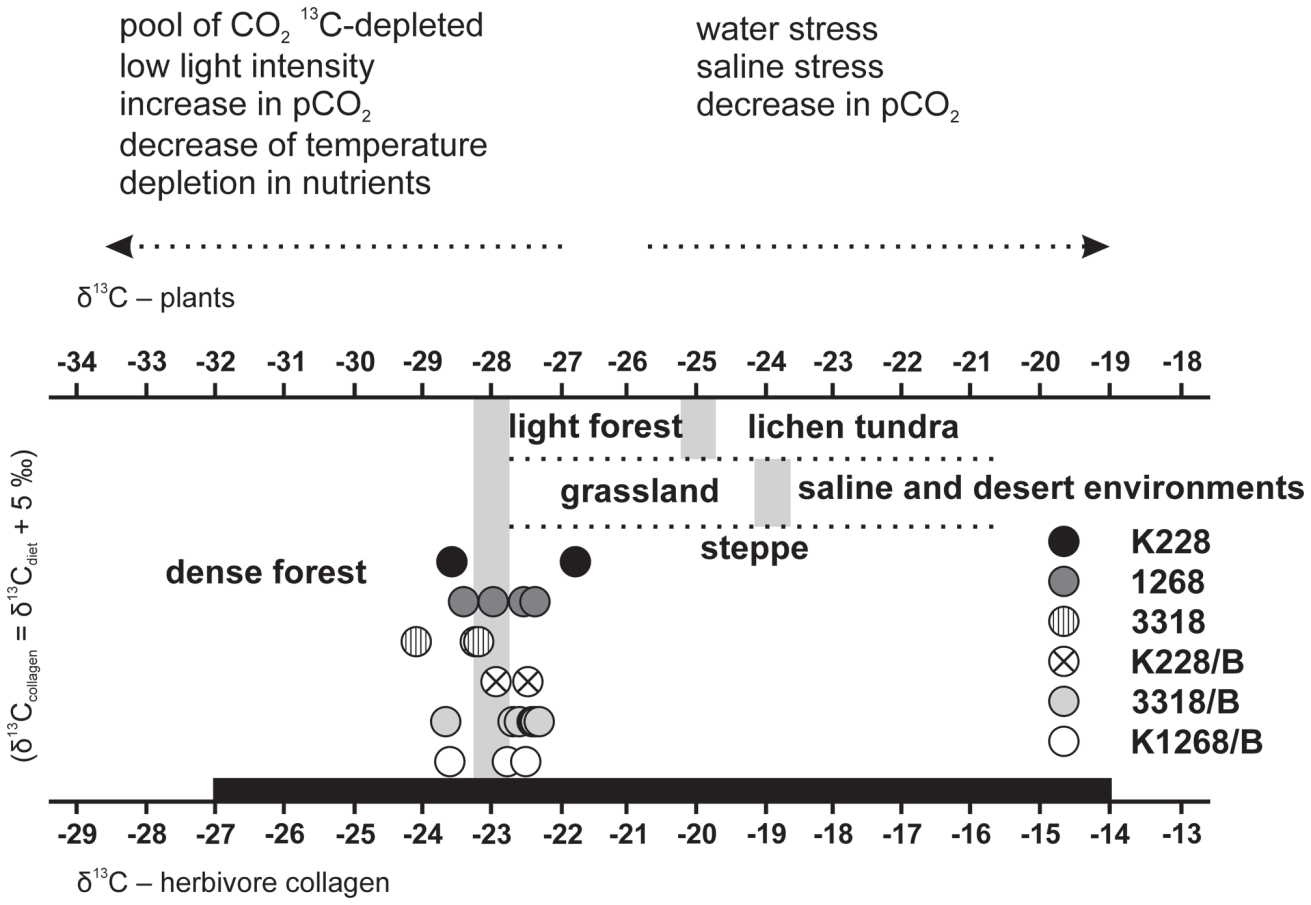
δ^13^C values obtained from horse hair excavated at the Medieval site Veselí na Moravě. The obtained values show that the individuals lived and moved in different types of environments. Environmental data was taken from Bocherens (2003), Nelson *et al.* (1986), Bocherens *et al.* (1994, 1996, 2000), Rodière et al. (1996).

**Figure 9:**
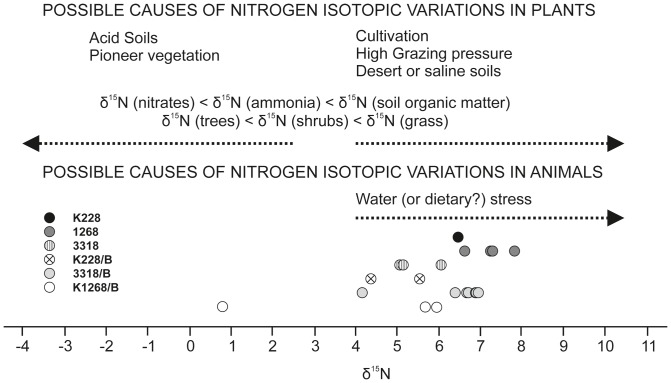
δ^15^N values obtained from horse hair excavated at the Medieval site Veselí na Moravě. The obtained values show that the individuals moved during the year in different environments. The environmental data was taken from the Bocherens (2003), Nelson *et al.* (1986), Bocherens *et al.* (1994, 1996, 2000), Rodière et al. (1996).

Three centimeter long sections of horse hair were analyzed. Since one millimeter of horse hair grows each day, this section represents one month of hair growth [42]. Surprisingly, the results of isotope analyses differ for each sample (Tab. 3), corresponding to different water stress and feeding provenance through time and for each individual. In the case of sample K228, a significant shift into negative values (2‰) for the δ13C measurement implies that the horse spent the first three months mainly in parks and meadows while the next three months correspond to wood vegetation (tree leaves, shrubs) (Fig. 8 and Fig. 9). Six centimeters of horse hair in sample 1268 were divided into 4 parts and they do not show any significant changes in feeding or environment. This individual lived or was fed (at least for the four month period tested) on meadow grass and/or in a sparsely wooded environment. Nine centimeter sections of horse hair in sample 3318, representing 3 months, changed significantly in the second and the third months. This change was tracked to a change in environment as well as in the type of feeding. The first change shows movement from meadow grasses to a wooded environment and then a significant movement back to the cultural landscape and meadows (Fig. 8 and Fig. 9). The horse hair of another individual (sample 3318B) measured 21 cm and was divided into 7 parts. Interestingly, no change in δ13C was detected between these seven sections. This individual was fed in a wooded environment. The last part of the sample shows significant improvement of environment and food and movement into a more humid environment (Fig. 8 and Fig. 9). Another individual (sample K1268B) provided a 9 cm long hair which was divided into three sections. The first two sections have isotopic values corresponding to a dry environment (Fig. 8 and Fig. 9). This individual was probably fed on hay. The third section of this sample shows a change to a woody, humid environment (Fig. 8 and Fig. 9). Sample K228B recorded two months of the individual's life and suggested that the environment and food did not change during this period. This individual lived in a humid environment and was fed on grass and tree leaves (Fig. 8 and Fig. 9).

### 6. Dendrochronology and dendrology

The average tree-ring series was dated using the Czech oak tree-ring chronology CZGES 2010 [43] to the year 1228. The period of tree felling could be established for sample no. 111 dated to 1230–1248 because it contained sapwood tree rings (ks); within the territory of the Czech Republic oak sapwood usually contains 5–25 tree rings depending on the age of the tree and location [44]. Sample No. 110 dated to the year 1202 did not contain sapwood tree rings (oak); therefore, we could only ascertain the year after which the tree was felled (Tab. 4).

**Table 4:** Dating of wood samples excavated within a Medieval bailey in Veselí nad Moravou.

Number of sample	Species	Length	End	Dating
110	elm	51+6ak	1191	after 1202
111	oak	107+2ks	1228	1230–1248
150	oak	46+19ak	1193	after 1217
159	oak	61+31ks	1196	1227–1241
162	oak	62+1ks	1222	1224–1244
205	oak	189+20ak	1188	after 1213

The individual construction components differ in the type of wood used. Poles supporting the roof and frames of the single entrances were made of oak. Doorstep from the wide northerly oriented entrance was made of oak and alder. The walls of the building were made from entwined sticks of unidentifiable broad-leaved wood, but the presence of a hazelnut suggests that this tree was used. Twenty vertical joists were also analysed. Nineteen were identified as oak wood and one as elm wood. Elm was also used for floor boards found in the eastern room. Wood found under the wooden floor was identified as elm and oak.

### 7. Zooarchaeology

Six species of animals were identified from 207 fragments (weighing a total of 2844.7 grams) recovered from the remediation (waste) layer and organic layers in the stable. The average weight of one fragment was 13.7 grams. Unburned fragments of mammal and bird bones were identified within remediation layers as well as within the organic part of the stabling. The following taxa were identified: Aves, *Anser domesticus*, Galliformes, *Gallus domesticus*, Mammalia, *Sus scrofa*, *Sus domesticus*, *Ovis/Capra*, *Bos Taurus*. Also taphonomic changes induced by humans and animals were identified. Butchering was evident on 29% of the fragments and tooth prints on 5% of the fragments. Weathering of bone surfaces was not significant.

## Discussion

### 1. Medieval horse stabling structures

Medieval stables are quite rare in the Central European archaeological record [45], probably because horses were often left outside on the pastures during summer and stables were usually used as a winter refuge only. The discovery of a Medieval stable in Veselí nad Moravou presents a unique opportunity to study not only architecture, but also stable maintenance and the type of horse alimentation.

The stable investigated in this project has two identified construction phases (Fig. 2). During the first phase (A) the stable served as a simple room with an entrance oriented to the NE. The structure was made from intertwined sticks, which was commonly used in Medieval times. Unlike the common types known from iconography [46], the stable from Veselí nad Moravou has plastered walls and the structure was quite robust. Such structures are also known from iconography, but rarely [47]. The structure itself was supported by posts and according to their positions, the building appears to have been 4–5 meters wide. Such joists can be easily made from common trees [48] [49]. If the spatial arrangement was equivalent to known data from 19th century, i. e. 1.3×3.5 m [50], the stable excavated in Veselí nad Moravou could have accommodated 7 horses.

The roof of the stable was not found. Stable roofs are not known from iconography, but ethnographic sources confirm their existence [51]. The 1.6 meter wide entrance was quite robust. In ethnography there are known doorways built from sticks, sealed up in winter by straw [51] [52]. The second phase of stable construction follows more or less the first phase. The differences are in the internal division of space where the second phase structure changes from one to three separate rooms. Also, two of the new rooms have a wooden floor. One of those rooms probably served for superior horses or pregnant mares, the second one probably as a preparation room. Preparation rooms are known from iconography [47]. The walls contained hazelnuts, which mean that this structure was built sometime in the late summer [53].

Other interpretations were also considered. For example, the structure may have been used as a storage room for feed, or for stabling of animals other than horses. Unfortunately there is not enough information about the differences between stabling structures used for different types of animals so we have only been able to compare our data with iconographic and etnographic works [46] [47] [51]. In interpreting the stabling deposits, the key features were the presence of organic matter and the internal structure of this part of the infill (compare with chapter 4.2.3) which is composed of organic matter as well as a mineralogical fraction interbedded within the organic matter. Such structure cannot form just from the deposition of organic matter; trampling during the deposition of organic matter must also have taken place [6]. The presence of horse hairs and artefacts typically used in horse husbandry contexts have also contributed to the final interpretation. If the structure was used as a storeroom, trampled horizons within the floor layers would be expected, instead of the organic matter that was actually found [54].

### 2. Maintenance practices in a Medieval stable

Maintenance practices of horse stables are not known from historical or archaeological sources. In the case of Veselí nad Moravou, we have opted to apply a sedimentological and micromorphological approach to determine the sediment composition and to track possible maintenance practices that were used. Analogous micromorphological studies concerning stabling or floor deposits from sites such as Veselí nad Moravou are difficcult to find because a micromorphological approach is still not widely used for such contexts. However, stabling has been identified and micromorphogically studied in tell sites by Matthews et al. [12]. These authors report stables, but in contrast to Veselí nad Moravou, usually without prepared surfaces. This could be due to different geological and geomorphological backgrounds. As in Veselí nad Moravou, the background is composed of easily erodible sediments with potential for a high water level. Accumulated deposits of tell stables are interbedded lenses of fragmented dung pellets with postdepositional changes typical mainly for organic staining, salts and bioturbation. In Veselí nad Moravou, the organic staining in calcium carbonate rich lenses of organic waste were preserved below the horse stabling, which is again also probably connected with the permeable geological units and the position within the alluvial zone of the Morava River.

Most of references concerning the study of object infillings are concerned with whether the infill originated naturally or anthropogenically [55] [56]. In the case of the Veselí nad Moravou stable, from the sedimentological and micromorphological view it is evident that the infilling originated antropogenically during remediation. Such examples are known for example from Hallstatt sunken houses [57] and from Viking houses [58]. Maintenance of Viking sunken houses in Iceland can be used as a comparison. The remediation layers have a sanitation effect and they increase the amount of easily erodible material on the surface. Floor surface erosion in Iceland is due to the volcanic background [58] [59]. In Veselí nad Moravou it is due to the sandy erodible background and the fact that during the removal of stabling the background is repeatedly removed as it adheres to the stabling. Due to such maintenance practices, at least 10 aggradation layers were preserved composed of waste from ovens and also common domestic waste as visible from the appearance of animal bones which were not burnt. On the other hand, such waste has a sanitation effect and and protects against hoof inflamation.

Preserved stabling was quite fresh due to the lack of ruderal species, i.e. waiting to be removed. Its pollen composition shows that the organic part of the stabling itself comes from the end of summer. But from an etnographic source [45] it is known that horses were stabled after the 16th of October (Feast Day of St. Havel). Until that time horses were pastured and lived outside 24 hours a day.

### 3. Horse alimentation in Medieval times and the origin of Veselí nad Moravou horses

Surprisingly, information about horse alimentation in Medieval Europe is limited [45] [60] [61]. It is a fact that during feudalism there was a shortage of adequate alimentation for animals, especially during the winter period [2] [3]. On the other hand the recent research in Siberia show, that livestok is able to fed outside the farm independently also during the winter periods, becuase the nutrition value of trees and shrubs is suficcient [62], but there must be evidently also limited number of animals per adequate area. We know from writen sources, that during summer, horses were kept in pasture or in the woods where they required minimal attention [63]. It is known that in the Late Middle Ages, it was common practice to collect branches with leaves suitable for feeding in the woods [2] [45]. Free pasture was possible during the 13th century and was enshrined in law - the so-called “the right of mare field”, which means that mares were allowed to move freely in the landscape during the time period before harvest [2] [45].

The oldest known record of purposeful horse feeding is from Ibrahím Ibn Jakúb from the mid-10th century. More information is available from Medieval England [64], mainly from accounting records [65]. In spite of the fact that 14^th^ century England differs culturally to Central Europe, the differences in alimentation appear to be small. Horses in Medieval England were fed on oats in winter and were pastured during summer. Also, hay and straw was commonly used as feed, with chaff, bran, horse bread, legumes (peas, beans, vetches) a minor component [65]. Horses in Bohemian countries were fed mainly on barley and later on oats [45]. Graus [2] claims that in the pre-Hussite period horses in Bohemian countries were fed oats, vetches and mélange. Draff was also used. Ethnographic sources reveal that draught horses in Southern Moravia were fed vetches and hay during summer because pasture was not sufficient [66]. During winter, horses were fed straw from vetches, lentils, peas or millet and hay. Also, rye and wheat straw as well as other fodder was used as feed [66].

The most frequently found types of macroremains in the stable sediments in Veselí nad Moravou are plants that typically grew in meadows, pastures, and harvested fields. Less frequently occurring macroremains belonged to rubble sites, weed of cereals and wood glade. It is difficult to differentiate between feeding and stabling material. Sources suggest wood glade was also used as feed [2] [45]. Veselí nad Moravou is situated on an alluvial plain, so the element rich alluvial grass was also probably used as stabling material and as a substitute for straw. On the other hand, we know from ethnographic sources that at the beginning of the 20th centrury, stabling with straw was less common than today. As fodder was in short supply, other materials may have been used [66]. Animal bone fragments were also detected; it appears likely that any material at hand was used as stabling material, but the overall amount of stabling material was small and most of the material described from Veselí nad Moravou probably comes from feed left by horses on the floor.

We do know for a fact that pasture was an important component of horse feed. The examined samples indicate Cynosurus pasture species (for example *Prunella vulgaris*). The presence of willow sprouts (*Salix sp*.), small blackberries (*Rubus sp*.), hornbeam nuts (*Carpinus betulus*) and fragments of acorns constitute evidence of horses being fed in wooded pastures. Cereals such as millet, oat and hemp seeds were also detected. Straw is commonly used as stabling material today. We know from ethnographic sources that it was often used as horse feed in Southern Moravia [66]. Such a spectrum of horse fodder is much greater than what we know from references for Central Europe or for England.

**Figure 10:**
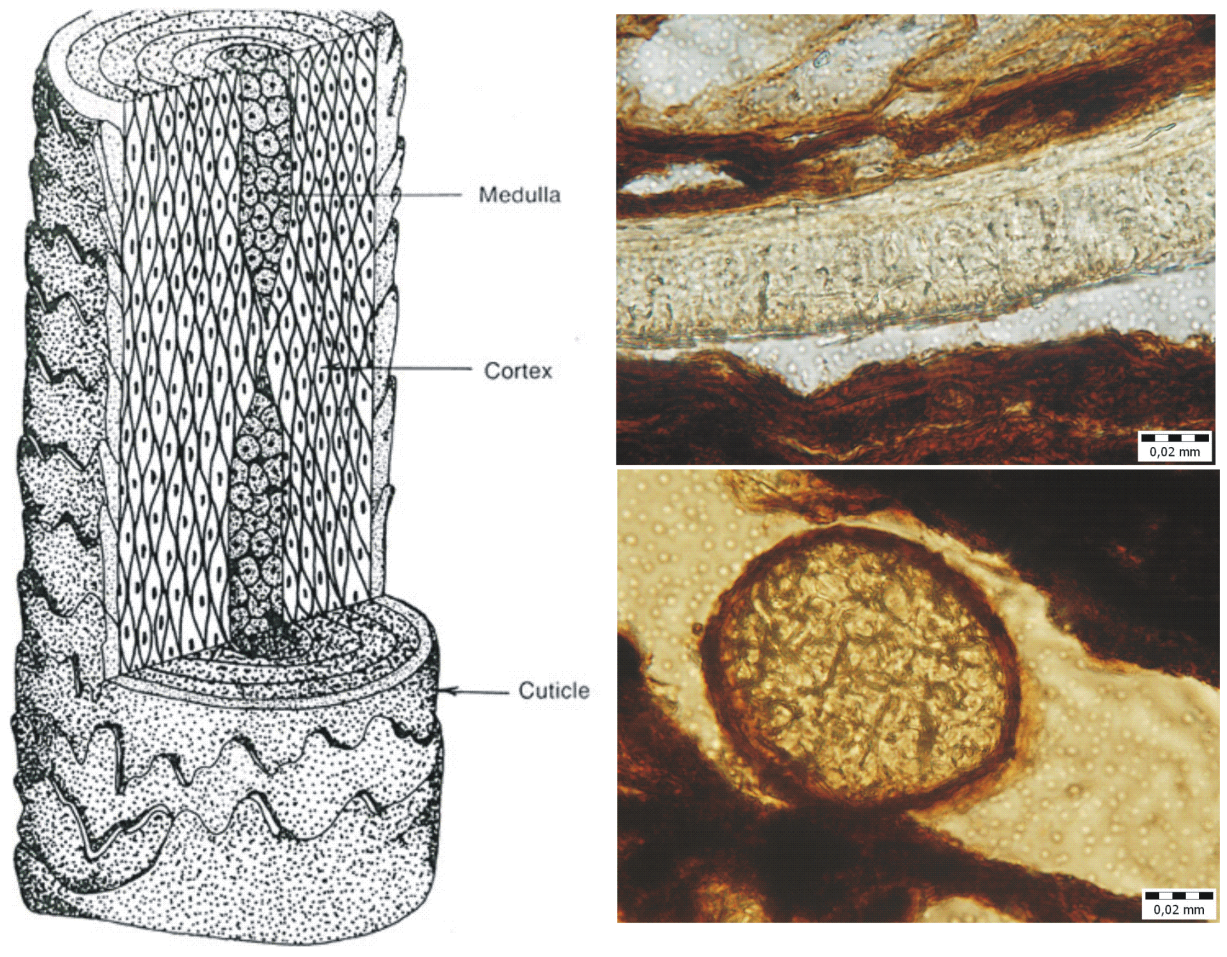
Micromorphology and the internal structure of horse hair.

It is also possible to obtain valuable information about horse feeding from analyses of C and N isotopes. The changes in these isotopes show environmental or seasonal changes [42] [67] [68]. Data about horse alimentation as interpreted from isotopic analyses of horse hairs (confirmed macroscopically and microscopically (Fig. 10) in large numbers), does not significantly differ from the horse alimentation generally presumed for Medieval horses in Central Europe [69]. Horse hair from Veselí nad Moravou shows significant changes in alimentation, or alternatively changes in the environment (Fig. 8 and Fig. 9) i.e. the transition between meadows to more humid woods and humid meadows (see Fig. 8 and 9). This transition can be interpreted as a seasonal change in alimentation (i.e. pasture during the vegetation season and feeding on hay during winter), or as structural differences in horse alimentation. For example, one of the horses often moved in the landscape where enough feed and water were available and moved between ecosystems. Another horse was kept in close surroundings of the castle and its opportunities for a varied diet were restricted.

The results published in this paper show that the fresh stable in Veselí nad Moravou was deposited in late summer. The horse population stabled in Veselí nad Moravou was quite varied. This inference can be explained in different ways. For example, the stable may have been used as temporary shelter for horses of peasants who came to work for the owner of the castle. Alternatively, the observed variability can correspond to different functions of the stable. If the stable was also servicing an inn, a number of courier houses could have been stabled there [70]. The third possible explanation for the presence of a large number of horses with different backgrounds in the one place is the possible presence of horses used in battle. In Medieval times, battle horses were commonly sourced from local inhabitants. One of the last references to Veselí nad Moravou dates to the year 1315 when Jan Lucemburský battled Matúš Čák Trenčianský and conquered the castle in Veselí nad Moravou during the late summer [71]. The excavated stabling could represent the last usage of this stable up to this event, but unfortunately it is difficult to confirm this hypothesis.

### 4. Medieval vegetation along the Morava River

The archaeobotanical record from stable deposits in Veselí nad Moravou also reflects the composition of surrounding vegetation, to varying degrees. As we are dealing with anthropogenic deposits, in interpreting them it is neccessary to take into account the fact that the species present in the stable were selectively introduced by humans (so they are not an accurate reflection of the actual vegetation structure) [72]. The deposits include two separate sources (airborne and humans & animals), so the information obtained from pollen, phytolith, macroremains and anthracological analyses must be interpreted separately.

The remediation layers contain burnt firewood which offers probably the best evidence of the structure of the surrounding vegetation [56]. Charcoal of oak forest and oak-hornbeam forest species, including hardwood forests of lowland rivers (*Quercus robur*, *Fraxinus angustifolia subsp. danubialis*, *Fraxinus excelsior*, *Acer campestre*, *Ulmus laevis*) dominate. Due to the presence of species such as *Rubus ssp*. or *Clinopodium vulgare*, i.e. indicators of woodland which can be interpreted as a form of forest management. Such type of forest produces firewood at the expense of structural wood that was probably imported from more distant and less populated areas. Beech charcoal was identified in the samples, but beech does not grow in a riverine environment. Species of willow-poplar forest (*Populus nigra*, *Populus alba*, *Salix alba*, *Salix fragilis*) and alder forest (*Alnus glutinosa*), i.e. wood coming from near the Morava River floodplain were also reported. This picture of riverine vegetation is quite different from an Early Medieval site in Roztoky near the Vltava River. This site is typical for fir, Scots pine, and beech, along with coppiced and light-demanding species [56].

The second type of deposit being interpreted in the stable infilling is the horse stabling which contains plant macroremains and wood fragments. These remains indicate the presence of alluvial forests, alluvial meadows and tilled fields. It seems to represent horse feed and stabling material rather than the surrounding vegetation which can be seen in natural organo-genic deposits, such as oxbow or peat [24].

Within the Medieval surroundings of Veselí nad Moravou we can postulate the existence of wet meadow/pasture communities (Morava River floodplain), in particular mesic meadows and to a smaller extent, dry and very dry grasslands. Meadow vegetation of the region (Lesser Carpathians) is already documented at least 2 500 years ago and is mainly the result of human activities [73]. The fields where the horse feed was harvested were fertile and rich in carbonates (for example the weed *Bupleurum rotundifolium*). The substrate of such fields is often loess as well as colluvial and alluvial deposits, so the horse feed was probably sourced locally. Even pest species are typical for warmer parts of Moravia (*Glaucium corniculatum*, *Chenopodium murale*) and correspond with the modern-day local situation [74].

## Conclusions

The following conclusions can be made:

1. Average age of the wood found in Veselí nad Moravou Medieval bailey was determined to date to the year 1228. The investigated stable had two construction phases. Whilst the older phase involved a simpler structure, in the subsequent phase, part of the structure had a wooden floor.

2. The infilling of the stable reflects maintenance practices. Ashy material was deposited to provide remediation effects and its aggradation also served to reduce difference between the interior and the exterior of the stables.

3. Well preserved horse stabling in the uppermost part of the horse stable structure infilling accumulated within a few months at the end of summer. The stabling material was composed mainly of wetland grasses and wooden annual shoots. Differences in horse alimentation identified within stabling were probably due to differences between individuals. Horses were fed on meadow grasses as well as woody vegetation, millet, oat and less often on hemp, wheat and rye.

4. The results of isotope analyses suggest that the horses accommodated in the stable came from varied backgrounds. Three possible explanations of stable usage can be inferred. It may have been used as a temporary stable for horses of peasants from outside the castle, courier horses or battle horses.

5. Within the Medieval surroundings of Veselí nad Moravou we can postulate the existence of communities of wet meadow/pastures (floodplain of the Morava River), common types of mesic meadows but also (to a smaller extent) dry and very dry grasslands. Woody vegetation includes oak and oak-hornbeam forests including hardwood forests of lowland rivers (*Quercus robur*, *Fraxinus angustifolia* subsp. *danubialis*, *Fraxinus excelsior*, *Acer campestre*, *Ulmus laevis*). Low forests (*Quercus robur*, *Carpinus betulus*) were probably cultivated.
